# Glutamate Supply Reactivates Ovarian Function while Increases Serum Insulin and Triiodothyronine Concentrations in Criollo x Saanen-Alpine Yearlings’ Goats during the Anestrous Season

**DOI:** 10.3390/ani10020234

**Published:** 2020-02-02

**Authors:** César A. Meza-Herrera, Hector P. Vergara-Hernández, Alicia Paleta-Ochoa, Alma R. Álvarez-Ruíz, Francisco G. Veliz-Deras, Gerardo Arellano-Rodriguez, Cesar A. Rosales-Nieto, Ulises Macias-Cruz, Rafael Rodriguez-Martinez, Evaristo Carrillo

**Affiliations:** 1 Unidad Regional Universitaria de Zonas Áridas, Universidad Autónoma Chapingo, Bermejillo, Durango 35230, Mexico; hector31_88@hotmail.com (H.P.V.-H.); ali_13_11_2008@hotmail.com (A.P.-O.);; 2 Instituto de Estudios de Posgrado, Universidad de Córdoba, 14014 Córdoba, Spain; 3 Departamento de Ciencias Médico Veterinarias, Universidad Autónoma Agraria Antonio Narro, Torreón, Coahuila 27054, Mexico; velizderas@gmail.com (F.G.V.-D.); gveterinarioarellano@gmail.com (G.A.-R.); rafael.rdz.mtz@gmail.com (R.R.-M.); 4 Facultad de Agricultura y Veterinaria, Universidad Autónoma de San Luis Potosí, San Luis Potosí 78321, Mexico; nieto_cesar@hotmail.com; 5 Instituto de Ciencias Agrícolas, Universidad Autónoma de Baja California, Mexicali 21705, Mexico; ulisesmacias1988@hotmail.com; 6 Instituto Tecnológico de Torreón, Torreón Coahuila 27170, Mexico; evaristocarrillo@yahoo.com.mx

**Keywords:** goats, anestrous, glutamate supply, ovarian reactivation, metabolic hormones

## Abstract

**Simple Summary:**

We evaluated the potential supplementation effect of glutamate upon reactivation of ovarian function and serum concentrations of insulin (INS) and triiodothyronine (T3) during the anestrous season in goats. Intravenous glutamate supply in yearling goats with a high level of very seasonal dairy goat breeds positively affected the reactivation of ovarian function during the anestrus season, which was positively linked to increases in both INS & T3 across time. Results denote the potential role of glutamate as a modulator not only of ovarian function, but also metabolic hormone synthesis. Such findings could be important in the design of reproductive strategies to attenuate seasonal reproduction in dairy goats, and may also embrace potential translational applications.

**Abstract:**

The possible effect of glutamate supplementation upon ovarian reactivation and serum concentrations of insulin (INS) and triiodothyronine (T3) in anestrous yearling goats was evaluated. Goats (n = 32, 12 mo., 26° North, 1117 m) with a similar live weight (LW) and body condition score (BCS) were blood sampled twice per week for two weeks (2 × 1 week × 2 weeks) to confirm the anestrus status (<1 ng P4/mL; RIA). Thereafter, goats were randomly assigned to either 1) Glutamate (GLUT; n = 16, LW = 27.1 ± 1.09 kg, 3.5 ± 0.18 units, IV-supplemented with 7 mg of glutamate kg^−1^ LW), or 2) Control (CONT; n = 16; LW = 29.2 ± 1.09 kg; BCS = 3.5 ± 0.18, IV saline). During the treatment period, 16 goats (eight/group) were blood sampled twice per week for six weeks. Such serum samples (2 × 1 week × 6 weeks) were quantified by their P4 content to evaluate the ovarian-luteal activity, whereas a sample subset (1 × 1 week × 6 weeks) was used to quantify their INS & T3 content to evaluate their metabolic status. Neither LW (28.19 kg; *p* > 0.05) nor BCS (3.51 units; *p* > 0.05) differed between treatments. Goats depicting ovarian reactivation favored the GLUT group (50 vs. 12.5%; *p* < 0.05). Neither INS (1.72 ± 0.15 ng mL^−1^) nor T3 (2.32 ± 0.11 ng mL^−1^) differed between treatments, yet a treatment x time interaction regarding INS & T3 concentration across time favored (*p* < 0.05) the GLUT group. The results unveil exogenous glutamate as an interesting modulator not only of ovarian reactivation, but of metabolic hormone synthesis.

## 1. Introduction

In small ruminants, mainly from temperate and subtropical regions, reproductive seasonality turns into a productive seasonality, generating serious marketing problems for goat products not only for producers, but also for industrializers and consumers, which demand a continuous goat product supply throughout the year [1,2]. At a global level, most goat production is carried out under low-input, marginal, rain-fed production systems, which commonly compromise the metabolic status of goats [1,3]. Whereas reproductive function is tightly modulated by metabolic status, a negative energy balance reduces sex steroids, diminishes pulsatile Luteinizing hormone (LH) secretion, and compromises both reproductive outcomes and economic return to producers [2,4,5]. Therefore, the design of a nutritional supplementation strategies to improve both reproductive and productive outcomes, especially in marginal production systems, should provide the opportunity to breed females out of season [1,3,4].

Although the activation of the hypothalamic-pituitary-gonad (HPG) axis has GnRH as its central triggering molecule, some important neurotransmitters may also be involved in order to align the HGP functionality to the main external environmental conditions [6,7,8]. Both kisspeptinergic and glutamatergic activation are closely involved in triggering the activity and functionality of this neuronal circuitry, which promotes the increase in the GnRH pulse [4,9,10,11,12]. Moreover, the excitatory amino acid glutamate has been regarded not only as the main neurotransmitter in the mammalian central nervous system (CNS) [9,10,13,14], but also as the modulator in the control of the HPG axis by affecting the GnRH pulse generator and, in turn, the gonadotropin release pattern [12,15,16]. Furthermore, due to its wide distribution of receptors in the different CNS synapses, glutamate has been recognized as the central regulator of a myriad of physiological processes [9,17]. Indeed, neurons within the hypothalamic ventral premammillary nucleus, co-expressing glutamate, and leptin receptors, may directly activate both kisspetinergic and GnRH neurons [10,18,19]. In addition, intracerebroventricular glutamate receptor agonist administration stimulates the secretion of GnRH and LH, proposing a role for glutamatergic neurotransmission outside the Kiss1 neuronal system [20]. Furthermore, both the medial and lateral nuclear compartments of the hypothalamus provide substantial input to the ventral tegmental area, whose afferents convey information about integrative physiological actions to ensure proper operation and regulation not only of reproduction, but also of feeding and energy expenditure. Such afferents denote a predominance of glutamatergic stimulation in most hypothalamic nuclei [21].

Previous studies conducted by our group demonstrated that glutamate acts as an important cue for sexual maturation in a glucose-independent pathway, while both insulin and triiodothyronine affected the establishment of puberty in goats in a significant fashion [22]. Later on, a positive influence of glutamate supplementation upon serum cholesterol concentrations was also observed around the onset of puberty in goats [23]. Yet, information regarding the relative changes in metabolic hormones that may serve as metabolic cues, reactivating the communication of the HPG axis and triggering the onset of a new estrous cycle in glutamate supplemented goats during the anestrus season, has not been elucidated. Building on such findings, this study aimed to evaluate the possible effect of glutamate supplementation upon reactivation of ovarian function and serum concentrations of the metabolic hormones insulin (INS) and triiodothyronine (T3) during the anestrous season in Criollo x Saanen-Alpine yearling goats.

## 2. Material and Methods

### 2.1. General

All the procedures, methods, and management of the experimental units used in this study were carried out in strict accordance with accepted guidelines for ethical use, care, and welfare of animals in research at international [24] and national [25] levels, with institutional approval reference number UACH-DGIP-REBIZA-IBIODEZA/15-510-400-2. 

### 2.2. Location, Environmental Conditions, Animals, and their Management 

The study was conducted at the Regional University Unit of Arid Zones, Chapingo Autonomous University (URUZA-UACH; 26° N, 103° W, at 1117 m), located in northern Mexico. The climate of the area is warm and dry, and the mean annual precipitation and temperature are 217.1 mm and 22.3 °C, respectively. The warmest month is June, with temperatures above 40 °C, whereas the coldest month is January, with the lowest temperature below 0 °C. Yearling goats (n = 32; 1/8 Criollo, 7/8 Saanen-Alpine, 11 mo. old), were fed a diet to meet 100% of their nutritional requirements [26]. Both the live weight (LW) and body condition score (BCS) were recorded every two weeks prior to feeding. BCS was evaluated in all animals by an experienced technician by palpation of the goat transverse and vertical processes of the lumbar vertebrae (L2 through L5) using a five-point scale (1 = very thin, 5 = very fat; [27]). Goats were fed twice per day with alfalfa hay (14% CP, 1.14 NEm Mcal kg^−1^) and silage corn (8.1% CP, 1.62 NEm Mcal kg^−1^) in the morning (07:00), and rolled corn (11.2% CP, 2.38 Mcal NEm kg^−1^) in the afternoon (18:00). Goats had *ad libitum* access to water, shades, and mineral salts under natural light conditions from March to April. The health status of all the experimental units was controlled by an experienced veterinarian during the whole experimental period, and no health problems were observed during the trial. Besides, efforts were made to minimize any possible discomfort in the experimental units.

### 2.3. Blood Sampling, Progesterone Quantification, and Confirmation of Anestrus Status

In early March, blood (10 mL) was collected (n = 32) twice per week during two weeks by jugular venipuncture prior to feeding. The blood was collected into sterile vacuum tubes (Corvac, Kendall Health Care, St. Louis, MO, USA) and allowed to clot at room temperature for 30 min. The serum was separated by centrifugation (1500× *g*, 15 min), decanted and collected in duplicate in polypropylene microtubes (Axygen Scientific, Union City, CA, USA), and stored at −20 °C until the hormonal analysis. The serum progesterone (P4) concentration was determined by radioimmunoassay (RIA) using a commercial RIA kit (Diagnostic Products, Los Angeles, CA, USA) validated for ruminant serum [28]. The intra- and inter-assay coefficients of variation (CV) were 9.9% and 12.4%, respectively. Whereas the average recovery was 94%, the sensitivity of the assay was 0.1 ng mL^−1^. Goats with P4 serum levels lower than 0.2 ng mL^−1^ were considered in anestrus status [29].

### 2.4. Experimental Design and Treatments

In mid-March, and after confirming the anestrus status of the experimental units, goats were randomly distributed in two experimental groups: (a) Glutamate supplementation (GLUT, n = 16; LW = 28.1 ± 1.0 kg, BCS = 3.5 ± 0.18 units), and (b) Control group (CONT, n = 16; LW = 29.2 ± 1.0 kg, BCS = 3.5 ± 0.18 units). The GLUT group received an intravenous infusion of 7 mg kg^−1^ LW of glutamate (C_5_H_9_NO_4_, Merck, Germany) twice per week throughout the experimental period. Meanwhile, the yearling goats of the CONT group received an intravenous application of saline to homogenize the conditions to which the goats of the GLUT group were exposed.

### 2.5. Intermittent Blood Sampling for Serum Progesterone, Insulin, and Triiodothyronine Levels and Ovarian Function

Once the goats were randomized into their respective treatments, an intermittent blood sampling (twice per week) was performed in 16 goats (eight/group) from mid-March throughout a period of six weeks. Serum samples within the experimental period were assessed in duplicate by their content of P4 (Diagnostic Products, Los Angeles CA, USA) by RIA. The test was modified and validated to be used in ruminant serum [28]. Whereas the intermittent, twice per week, blood samples were considered to evaluate serum P4, those serum samples collected every two weeks were used to quantify INS and T3 concentrations.

The serum T3 concentrations were determined in duplicate by solid-phase RIA using components of a commercial kit. The kit utilized antibody-coated tube technology, while the assay was performed without prior extraction of T3 from the serum (Diagnostic Products, Los Angeles, CA, USA [30]. Whereas the intra- and inter-assay CV values for T3 quantification were 0.66% and 6.96%, respectively, the sensitivity of the assay was 0.1 ng mL^−1^. The sources for T3 iodination and the standards were NEX 110 (New England Nuclear, Boston, MA, USA) and T2877 (Sigma, St. Louis, MO, USA), respectively. Peripheral insulin concentrations were determined in duplicate in blood serum using components of a commercial solid-phase RIA kit (Diagnostic Products, Los Angeles, CA, USA) according to the outline proposed by Sanson and Hallford [31]. The intra- and inter-assay coefficient of variation value for insulin quantification were 4.26% and 4.09%, respectively, with a detection limit of 0.2 ng mL^−1^. Endocrine analyzes were performed at the Department of Animal Science, New Mexico State University, USA.

**Figure 1:**
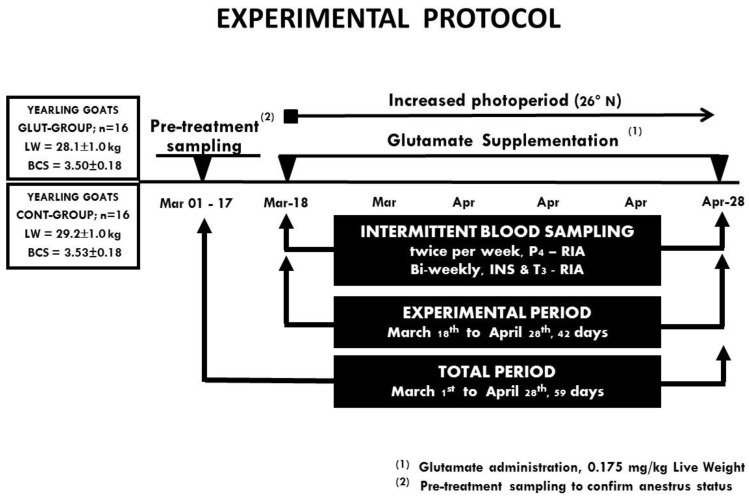
A schematic representation of the experimental protocol, including treatment groups and pre-treatment sampling to confirm anestrous status and the intermittent blood sampling to quantify serum progesterone, insulin, and triiodothyronine in Criollo x Saanen-Alpine yearling goats (n = 32) supplemented with Glutamate (GLUT) or control (CONT) under natural photoperiodic conditions during the anestorous season (March to April) in northern Mexico (26°LN). Note: Intermittent blood samplings were performed to evaluate serum progesterone (P4; twice per week), while insulin (INS) and triiodothyronine (T3) were collected every two weeks. Hormones were quantified using radioimmunoassay. Ovarian activity was defined to occur when serum P4 concentrations were ≥1.0 ng/mL.

The reactivation of ovarian function was confirmed in both experimental groups based on the P4 serum profiles. For each goat, a serum P4 level ≥ 1 ng mL^−1^ in two consecutives samples was indicative of ovulation (luteal activity) during the anestrus season (March—April). Therefore, the effect to offer or not supplemental glutamate upon reactivation of ovarian function, as well as serum INS and T3 concentrations, was evaluated. A schematic representation of the main activities performed during the experimental period are depicted in Figure 1.

### 2.6. Statistical Analyses 

The response variables LW and BCS, and serum P4, INS, and T3 concentrations throughout the experimental period were determined by split-plot ANOVA for repeated measures across time. The models included treatment in the main plot, which was tested using animal within treatment as the error term. Time and the time x treatment interaction were included in the subplot and were tested using the residual mean square [32]. In the case of a significant treatment effect, mean separations were achieved using the PDIFF option of the PROC-GLM. The proportion of goats depicting or not depicting puberty was compared with a chi-square test. All the analyses were computed using the procedures of SAS (SAS Inst. Inc. V9.1; Cary, NC, USA). Results are expressed as least-square means and standard errors and evaluated at the significance level of *p* = 0.05.

## 3. Results

**Figure 2:**
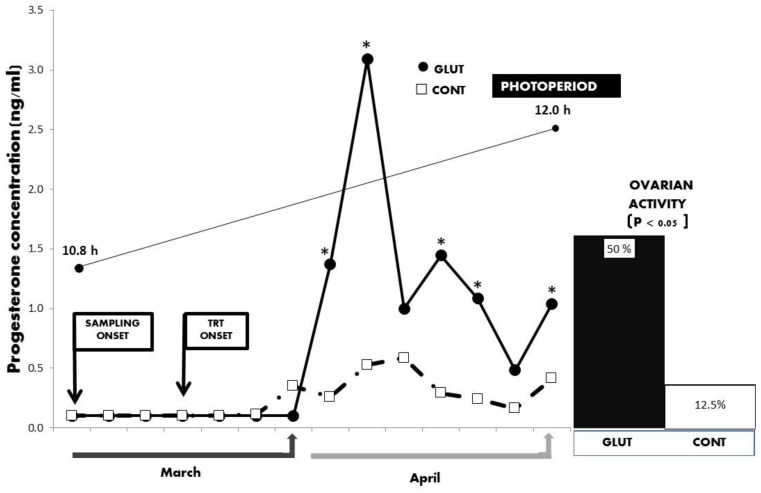
Serum concentrations of progesterone (ng/mL) between treatments across time in Criollo x Saanen-Alpine yearling goats (n = 16; 8/group) supplemented with glutamate (GLUT) or control (CONT) under natural photoperiodic conditions during the anestrous season (March to April) in northern Mexico (26°LN). Note: Whereas the onset of both blood sampling and treatments are indicated, a line denoting the prevailing photoperiod during the experimental period is also included. Superscripts indicate differences (*p* < 0.05) between treatments within time.

**Figure 3:**
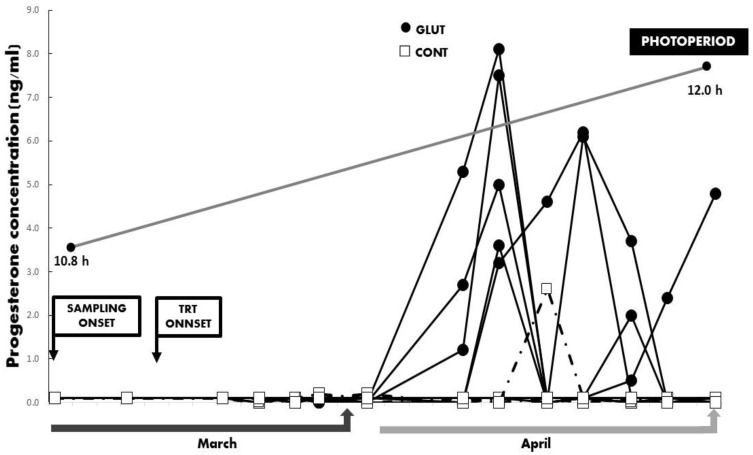
Serum concentrations of progesterone (ng/mL) were collected across time in Criollo x Saanen-Alpine yearling goats (n = 16, 8/group). Experimental groups consider glutamate (GLUT) and control (CONT) under natural photoperiodic conditions during the anestrous season (March to April) in northern Mexico (26°LN). Note: Whereas the onset of both blood sampling and treatments are indicated, a line denoting the prevailing photoperiod during the experimental period is also included.

Initial general averages for LW and BCS were 28.6 ± 1.0 kg and 3.5 ± 0.18 units, with respective values at the end of the experimental period of 33.5 ± 0.9 kg and 3.4 ± 0.16 units. No differences (*p* > 0.05) between treatments were observed for LW and BCS along the experimental period. Besides, while the total average concentration and the serum concentrations across time for serum progesterone differed between treatments favoring the GLUT groups (*p* < 0.05; 2.43 ± 0.66 vs. 0.95 ± 0.65 ng mL^−1^), a correspondent increased percentage for ovarian reactivation during the anestrus season was observed in the GLUT group (*p* = 0.05; 50 vs. 12.5%) (Figure 2 and Figure 3).

**Figure 4:**
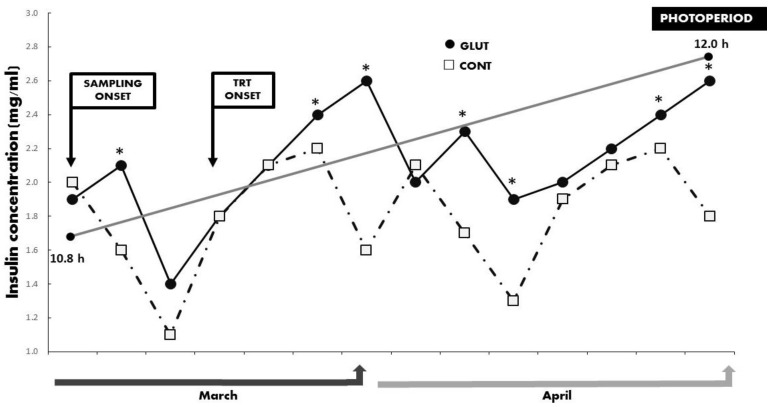
Serum concentrations of insulin (mg/mL) between treatments across time in Criollo x Saanen-Alpine yearling goats (n = 16; 8/group). Experimental groups consider glutamate (GLUT) and control (CONT) under natural photoperiodic conditions during the anestrous season (March to April) in northern Mexico (26°LN). Note: Whereas the onset of both blood sampling and treatments are indicated, a line denoting the prevailing photoperiod during the experimental period is also included. Superscripts indicate differences (*p* < 0.05) between treatments within time.

**Figure 5:**
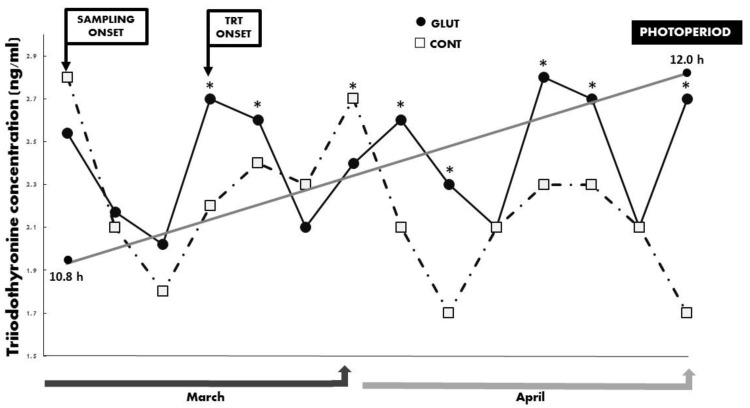
Serum concentrations of triiodothyronine (ng/mL) between treatments across time in Criollo x Saanen-Alpine yearling goats (n = 16; 8/group). Experimental groups consider glutamate (GLUT) and control (CONT) under natural photoperiodic conditions during the anestrous season (March to April) in northern Mexico (26°LN). Note: Whereas the onset of both blood sampling and treatments are indicated, a line denoting the prevailing photoperiod during the experimental period is also included. Superscripts indicate differences (*p* < 0.05) between treatments within time.

Regarding the quantified metabolic hormones, no differences (*p* > 0.05) were observed regarding the mean serum concentrations for INS (1.73 ± 0.15 ng mL^−1^) and T3 (2.33 ± 0.11 ng mL^−1^). Yet, a significant treatment x time interaction (*p* < 0.05) occurred between treatments for INS and T3 across time, and such differences favored the GLUT group (Figure 4 and Figure 5). The prevailing photoperiod during the experimental period was also included in Figure 2, Figure 3, Figure 4 and Figure 5. Finally, and quite interestingly, no differences for LW (*p* > 0.05; GLUT: 28.25 ± 0.84 kg & CONT: 28.87 ± 0.95 kg) nor for BCS (*p* > 0.05; GLUT: 3.41 ± 0.14 units & 3.51 ± 0.16 units) occurred between treatments in those goats depicting or not depicting reactivation of ovarian activity during the experimental period (anestrus season-increased photoperiod).

## 4. Discussion

Our working hypothesis proposed a positive effect of glutamate supplementation upon reactivation of reproductive function in yearling goats during the anestrus season. Such a hypothesis was not rejected because our results confirm an increased reactivation of the reproductive function of yearling goats supplemented with glutamate at this latitude (26° N). Moreover, such a neurophysiological scenario could be related to the observed increases in both serum INS and T3 across time. Although there is an intricate circuitry that controls the inhibition of GnRH release during the anestrus season in species classified as seasonal reproducers, our results delineate a potential action of glutamate as a modulating molecule capable of diminishing the negative feedback that estradiol exerts upon hypothalamic centers that abolish the hypothalamic release of GnRH during the anestrus season [4,33]. The precise site of glutamate action along the HPG axis to potentially decrease such negative retroaction of estradiol upon GnRH neurons is still elusive, and further studies on this respect are therefore warranted.

Glutamate has been defined as the main excitatory neurotransmitter in the mammalian brain [14,34], whereas glutamatergic and kisspeptinergic neurons have been associated with the activation of GnRH neurons, especially as important players in the establishment of the preovulatory GnRH surge [4,7,10]. Certainly, a decrease in the hypothalamic release of glutamate in the mediobasal and anterior preoptic area was related to a reduction in the GnRH, LH, FSH, and steroid concentrations, compromising the normal function of the HPG axis [35]. In addition, previous reports by our group defined a positive effect of glutamate supplementation upon reproductive outcomes not only in peripuberal goats [22,23], but also in adult goats [36]. Yet, such positive outcomes of glutamate administration upon reproductive performance occurred under photo-inductive, short-day photoperiods, while present upshots were generated under photo-inhibitory, long-day photoperiods (i.e., anestrous season) at this latitude.

Both live weight and body condition score are a consequence of metabolic changes, which are directly involved with reproductive success [4]. Any failure to assure the alignment between metabolic status and the activity of the HPG axis may compromise not only the productive or reproductive processes (i.e., timing ovarian function, fertilization, and gestation), but also the offspring’s survival and under extreme metabolic scarcity conditions and the viability of dams [37]. Such alignment found its roots in that a complex circuitry converges at the hypothalamic level, regulating both metabolic state mainly in the preoptic area, as well as reproductive function, primarily in the ventromedial nucleus and the arcuate nucleus [4]. Yet, in the current study, no differences in LW and BCS were observed between treatments, neither at the binging nor at the end of the experimental period. Moreover, even within treatment, no differences in LW and BCS occurred in those goats depicting or not depicting ovarian function. Therefore, other metabolic cues besides LW and BCS must be related to the reactivation of ovarian activity during the anestrus season in goats supplemented with glutamate.

Previous studies in peripubertal goats demonstrated a positive effect of glutamate supplementation upon metabolic status, considering both INS and T3 as endocrine markers [22]. Whereas insulin tightly modulates peripheral glucose homeostasis through the stimulation of not only glucose uptake, but also of either its oxidation or storage [4,5], the central role of insulin as a regulator of reproductive function has been recognized by acting as an essential molecule linking metabolic status and reproductive outcomes [5,38]. The same is true regarding T3 action upon metabolic and reproductive outcomes. Whereas a fundamental role of T3 upon growth and development besides the regulation of metabolism was previously reported [39], thyroid hormones (TH; T3 & T4) have been linked to different aspects to reproductive function, with both pro-gonadal and anti-gonadal actions being postulated [40,41]. Interestingly, glutamate and other glutamate receptor agonists (i.e., kainic acid, domoic acid, and NMDA) have promoted increases in both thyroid-stimulating hormone and TH, unveiling a key role of the excitatory amino acid system upon activation of the pituitary-thyroid axis [42,43]. Moreover, a T3-dependent testosterone-increased release was observed in male goats during puberty, suggesting a positive role of T3 upon Leydig cell steroidogenesis [44]. In females, mRNA alpha and beta receptors have been reported in granulosa cells of preovulatory follicles. Hence, a direct TH action upon the regulation pituitary-ovarian axis could be expected, considering such action of granulosa nuclear-TH receptors upon follicular growth and maturation [45]. Moreover, while T3 in combination with FSH increases granulosa cell proliferation and inhibits granulosa cell apoptosis, T3 has also been recognized as a biological amplifier of the stimulatory action of gonadotropins upon ovarian function [46].

The results of this study establish an important role of glutamate in the interpretation of environmental information (i.e., anestrus season) and the subsequent modulation of the neuroendocrine reproductive axis, in that glutamate supplementation promoted a positive effect upon the reactivation of ovarian function, which was escorted by an increased release of INS and T3 across time in yearling goats exposed to long-day photoperiods during the natural seasonal anestrus (26° N). 

Our results suggest that, in yearling goats supplemented with glutamate, the negative feedback exerted by estradiol upon the hypothalamic-pituitary axis, which inhibits the activation of the LH pulse generator, was significantly reduced. Certainly, during seasonal anestrus, an inhibition of reproductive function caused by the negative feedback of estradiol upon hypothalamic centers has been observed [4]. Therefore, our results suggest that the responses generated by the supplementation of glutamate may have sponsored a reduced sensitivity to the negative feedback of estradiol upon GnRH release, which may occur due to a possible downregulation of the genes involved in the hypothalamic mRNA expression of estradiol receptors. Such a scenario may have caused hypothalamic desensitization to the estradiol inhibition and, in parallel, promoted the reactivation of the HPG axis in most of the glutamate-supplemented yearling goats in the middle of the natural anestrous season. Moreover, while insulin and IGF-1 stimulate not only estradiol secretion by granulosa cells, but also follicular cell proliferation, the GH-pulsatile pattern varies in a steroid-dependent fashion affecting, in turn, IGF-1 secretion throughout the estrous cycle [47]. Merging such findings with our results, it is tempting to suggest that the observed P4 and insulin release in our study could be related to a direct glutamate effect. It may be also related to changes in some metabolic hormones throughout the estrous cycle, which, as an interesting pending assignment, deserves to be elucidated. 

Therefore, glutamate supplementation to anestrus goats stands out as an interesting alternative nutritional management tool in order to reduce reproductive seasonality, even in crossbred goats with high incorporation of highly seasonal dairy goat genes, like those observed in the Comarca Lagunera, Mexico. This would allow a better breeding distribution across the year, generating, in parallel, the possibility to offer goat products (i.e., milk and meat) in a less seasonal fashion, which is a reproductive-productive scenario that would benefit to producers, industrializers, and consumers.

## 5. Conclusions

To conclude, intravenous glutamate supply in yearling goats with a high degree of seasonal dairy goat genes positively affected the reactivation of ovarian function during the anestrus season, a reproductive scenario positively linked to increases in both insulin and triiodothyronine across time. Since no differences in LW nor BC occurred between experimental groups, such reproductive and endocrine outcomes suggest that glutamate supply may be involved in the attenuation of the negative feedback exerted by estradiol upon the HPG axis during the natural anestrous season. Results also denote the potential role of glutamate as a modulator not only of ovarian function reactivation, but also metabolic hormone synthesis, which could be of importance in the design of nutritional-reproductive strategies to attenuate seasonal reproduction in goat production systems and potentially important to other goat production systems or animal industries, as well as potentially interesting from a translational standpoint.

## Data Repository Resources

None of the data were deposited in an official repository, yet, information can be available upon request.
